# Application of hepatic lobe hyperplasia techniques in the treatment of advanced hepatic alveolar echinococcosis: a single-centre experience

**DOI:** 10.1186/s12893-022-01864-w

**Published:** 2022-12-06

**Authors:** Qiang Guo, Maolin Wang, Kai Zhong, Jialong Li, Tiemin Jiang, Bo Ran, Paizula Shalayiadang, Ruiqing Zhang, Talaiti Tuergan, Tuerganaili Aji, Yingmei Shao

**Affiliations:** 1 Department of Hepatobiliary and Echinococcosis Surgery, Digestive and Vascular Surgery Center, The First Affiliated Hospital of Xinjiang Medical University, Urumqi, 830054 Xinjiang China; 2 Clinical Medical Research Center of Echinococcosis and Hepatobiliary Disease of Xinjiang Uygur Autonomous Region, Urumqi, 830054 Xinjiang China

**Keywords:** Hepatic alveolar echinococcosis, Selective portal vein embolization, Two-stage hepatectomy, Future liver remnant volume

## Abstract

**Background:**

This study was designed to investigate clinical efficiency and application indications of hepatic lobe hyperplasia techniques for advanced hepatic alveolar echinococcosis (AE) patients.

**Methods:**

A retrospective case series covering 19 advanced hepatic AE patients admitted to the First Affiliated Hospital of Xinjiang Medical University from September 2014 to December 2021 and undergoing hepatic lobe hyperplasia techniques due to insufficient remnant liver volume were analyzed. Changes of liver function, lesions volume, remnant liver volume, total liver volume before and after operation have been observed.

**Results:**

Among the patients, 15 underwent portal vein embolization (PVE). There was no statistical difference in total liver volume and lesions volume before and after PVE (*P* > 0.05). However, the remnant liver volume was significantly increased after PVE (*P* < 0.05). The median monthly increase rate in future liver remnant volume (FLRV) after PVE stood at 4.49% (IQR 3.55–7.06). Among the four patients undergoing two-stage hepatectomy (TSH), FLRV was larger than that before the first stage surgery, and the median monthly increase rate in FLRV after it stood at 3.34% (IQR 2.17–4.61). Despite no statistical difference in total bilirubin (TBil), albumin (Alb), alanine aminotransferase (ALT), aspartate aminotransferase (AST) and gamma-glutamyl transpeptidase (GGT) in all patients with PVE, four patients who underwent TSH showed a decrease in ALT, AST and GGT. During the waiting process before the second stage operation, no serious complications occurred in all patients.

**Conclusions:**

For patients suffering from advanced hepatic AE with insufficient FLRV, PVE and TSH are safe and feasible in promoting hepatic lobe hyperplasia.

## Introduction

Echinococcosis is a parasitic zoonosis caused by cestodes of the genus *Echinococcus* [1, 2]. Alveolar echinococcosis (AE) is the most lethal and has gradually become an emerging disease in recent years. About 18,000 new cases are added every year in the world, with an incidence rate of 0.03 to 1.2 per 100,000 in epidemic areas, mainly distributed in countries in the northern hemisphere, such as France, Germany, Switzerland, Austria and China, of which more than 90% occur in China [3–5]. AE has been ranked as the second most important food-borne parasitic disease worldwide by the Food and Agriculture Organization of the UN and WHO [6, 7]. Notably, water could be a potential source of *E. multilocularis* infection in humans and animals in endemic areas [8, 9]. In the life cycles of *Echinococcus multilocularis,* human beings serve as the intermediate host. The most common organ involved is the liver (about 97%), which can cause hepatic AE [10]. Radical surgical resection of the lesions is the optimal choice for patients with hepatic AE [4]. However, hepatic AE lesions showed tumor like invasive growth, tended to invade most of the liver and important vessels at the advanced stage and even led to extrahepatic metastasis accompanied by adjacent organ invasion forcing many to turn to liver transplantation as the final treatment [11, 12]. Ex vivo liver resection and auto transplantation (ELRA) was first applied to treat advanced hepatic AE patients by Wen Hao's team in 2010 [13]. With no need to consider the graft source, this surgical procedure widens the indications of radical resection.

However, sufficient future liver remnant volume (FLRV) serves as the premise for ELRA [14], while insufficient remnant liver volume is commonly witnessed in patients with liver cancer. Consequently, scholars at home and abroad have successively proposed such methods as portal vein embolization (PVE), two-stage hepatectomy (TSH) and associating liver partition and portal vein ligation for staged hepatectomy (ALPPS), striving to provide surgery possibilities for patients with insufficient remnant liver volume and reaping good clinical efficacy. At present, there are many studies on the promotion of liver lobe proliferation concerning treatment of hepatic malignant tumors at home and abroad compared to few reports of its application to hepatic AE. This study analyzed the clinical data of 19 consecutive advanced hepatic AE patients with insufficient remnant liver volume who were treated with lobar proliferation promotion techniques, and explored the application and efficacy of such techniques in the treatment of advanced hepatic AE.

## Materials and methods

### Settings of the study

This retrospective study covered consecutive patients admitted into the First Affiliated Hospital of Xinjiang Medical University from September 2014 to December 2021. These patients were diagnosed with advanced hepatic AE but could not undergo one-stage radical resection due to insufficient volume of remnant liver. The patients studied were selected according to the following criteria: (i) hepatic AE was exactly diagnosed according to the expert consensus on diagnosis and treatment of hepatic cystic and alveolar echinococcosis (2019 edition); (ii) the ratio of FLRV to total liver volume (TLV) was less than 30% (it was less than 40% in patients with chronic hepatitis), and one-stage surgical removal of AE lesions was impossible; (iii) liver functions were classified into Child–pugh grade A or grade B, or their liver functions were classified into grade A or grade B after treated by percutaneous transhepatic cholangial drainage (PTCD); (iv) the patients had undergone PVE or TSH. On the contrary, patients were excluded from the study in the following cases: (i) combined with dysfunction of such important organs as heart, brain, lung and kidney, surgical operation may not be tolerated; (ii) patients were to receive liver transplantation or living donor liver transplantation; (iii) patients who received palliative therapy; (iv) patients accompanied with severe portal hypertension and portal vein thrombosis.

### Study methods

#### Preoperative examination and evaluation

Abdominal ultrasound, computed tomography angiography (CTA), magnetic resonance imaging (MRI), as well as magnetic resonance cholangiopancreatography (MRCP) were performed in all patients to assess the location of AE lesions and invasion of main vascular structures. Moreover, digital three-dimensional (3D) reconstruction helped to fully understand the spatial relationship among lesions, intrahepatic vessels and biliary ducts, which was also valuable for designing virtual surgical resection scheme, evaluating FLRV and determining whether surgical removal was tolerated or not. All patients received functional liver reserve test, and surgical contraindications were evaluated based on cardiopulmonary examination and routine examination. Resectability and specific procedures of PVE or TSH were carefully determined by a multidisciplinary team (MDT).

### Surgical procedure

#### PVE

**Fig. 1:**
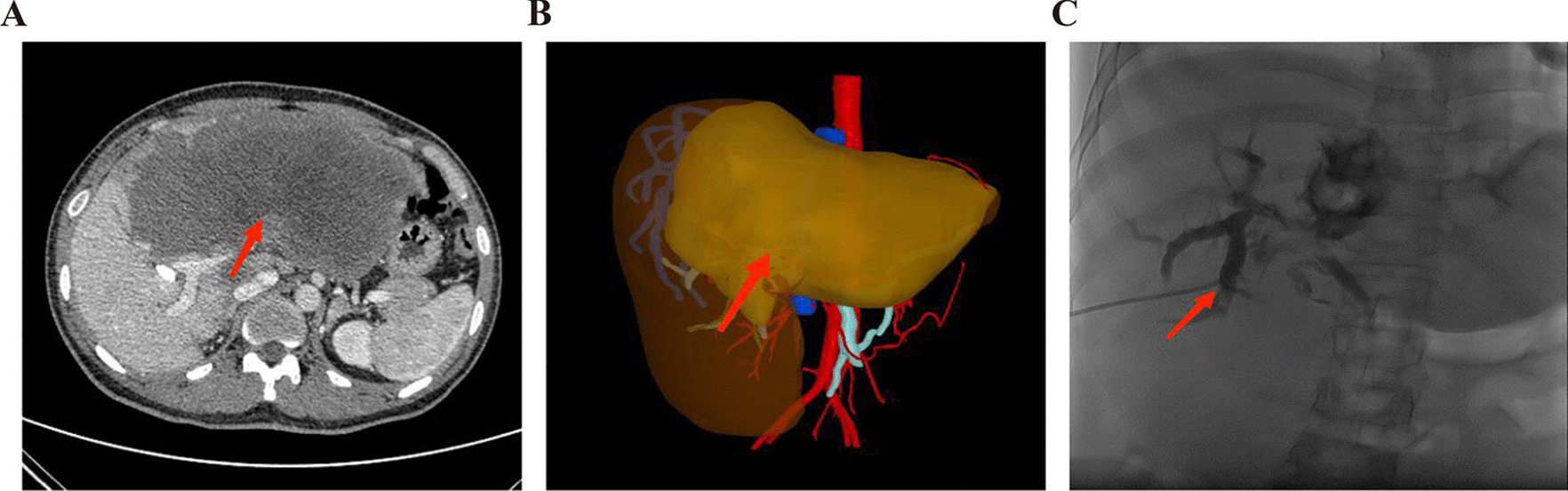
Representative preoperative images of PVE. **A** typical CT imaging before PVE; **B** digital 3D reconstruction before PVE (FLRV/TLV < 30%); **C** PTCD puncture and right posterior lobe biliary drainage

**Fig. 2:**
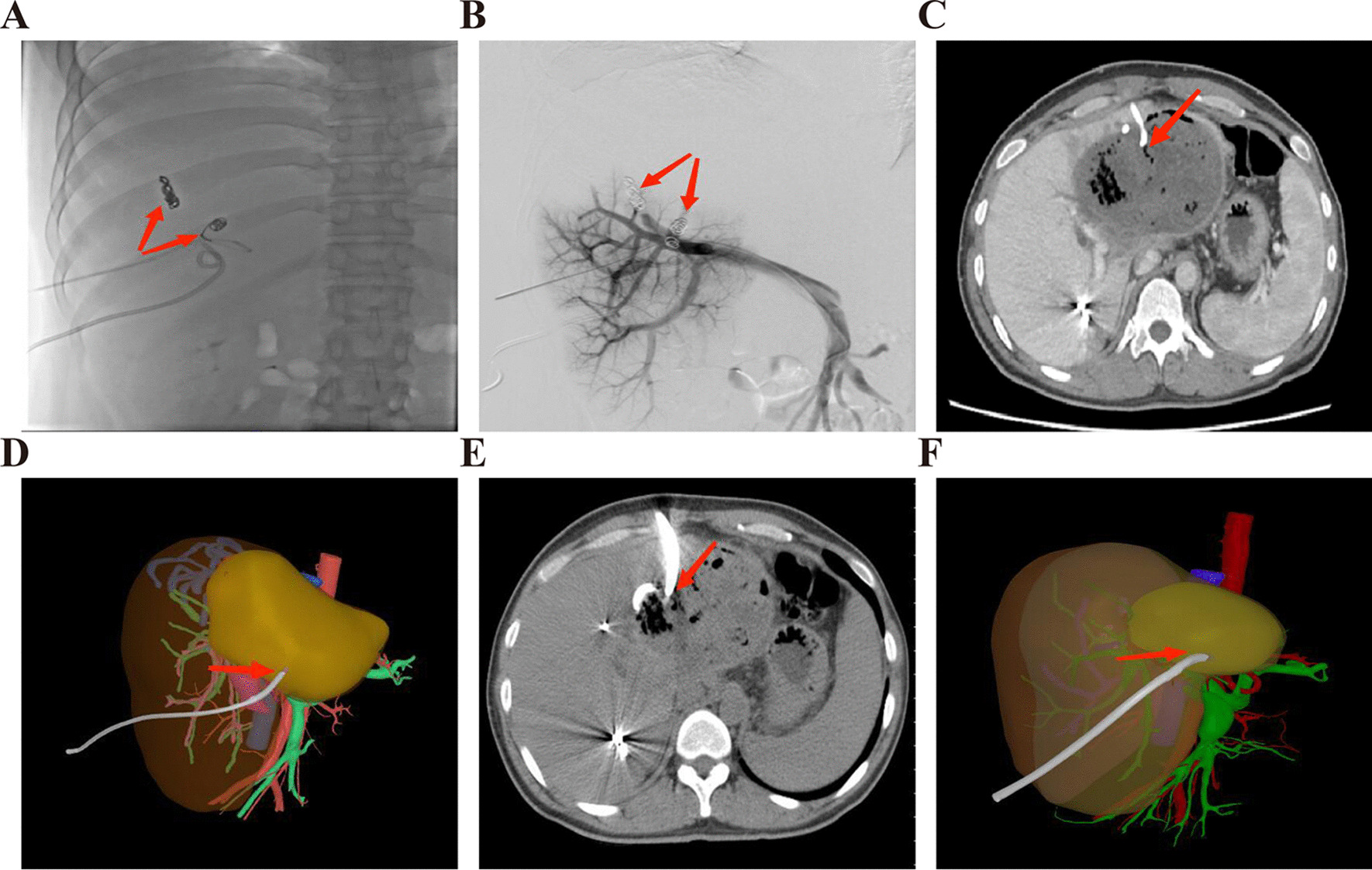
PVE and AE liquefaction cavity puncture. **A**, **B** successful embolization of right anterior branch of portal vein; **C** puncture and drainage of AE liquefaction cavity three months after PVE; **D** digital 3D reconstruction three months after PVE (FLRV/TLV < 30%); **E** the AE lesion reduced significantly six months after PVE; **F** digital 3D reconstruction six month after PVE (FLRV/TLV > 30%)

**Fig. 3:**
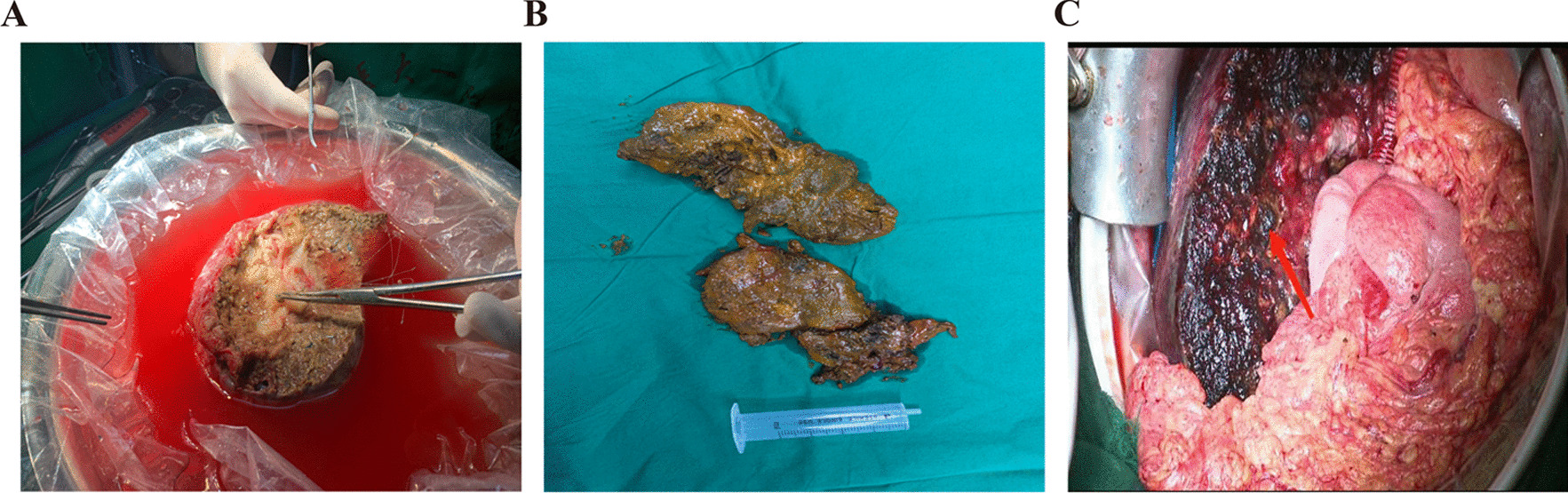
ELRA procedures. **A** ex vivo repair of the graft liver segment; **B** atrophic liver AE lesions; **C** successful transplantation of the repaired graft liver segment

Percutaneous hepatic puncture and portal vein intubation were applied for PVE. After anesthesia took effect, the unaffected side branch of portal vein was punctured under the guidance of digital subtraction angiography (DSA) before the catheter was inserted into the main portal vein along the guide wire. When there was no obvious stenosis and thrombus variation in the main portal vein and healthy side branches, the catheter sheath and catheter were introduced into the affected side branch of portal vein along the guide wire. After embolization with gelatin sponge particles and coils, portal venography was performed again to confirm complete embolization of the affected collateral branches of portal vein, and the catheter and catheter sheath were pulled out. While the healthy side liver volume was proliferated enough to perform the second stage surgery, radical surgery would be practiced. Representative preoperative and intraoperative pictures for these patients were shown in Figs. 1, 2, 3.

#### TSH

**Fig. 4:**
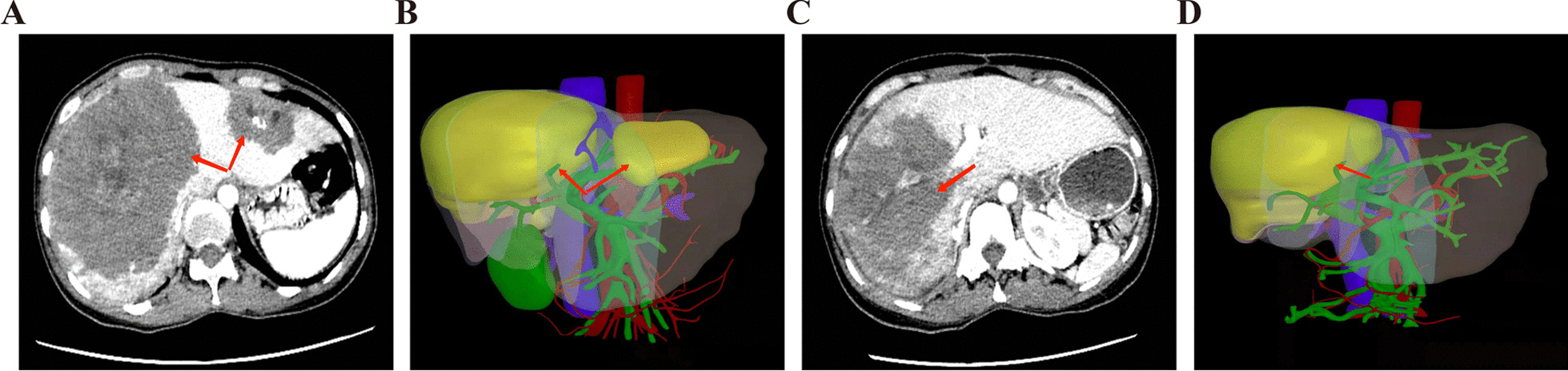
After TSH treatment, TSH2 patient, with FLRV/TLV > 30%, was able to receive ELRA treatment. **A** the first stage preoperative CT image; **B** the first stage preoperative digital 3D reconstruction of liver (FLRV/TLV < 30%); **C** CT image before the second stage operation; **D** the second stage preoperative digital 3D reconstruction of liver (FLRV/TLV > 30%)

**Fig. 5:**
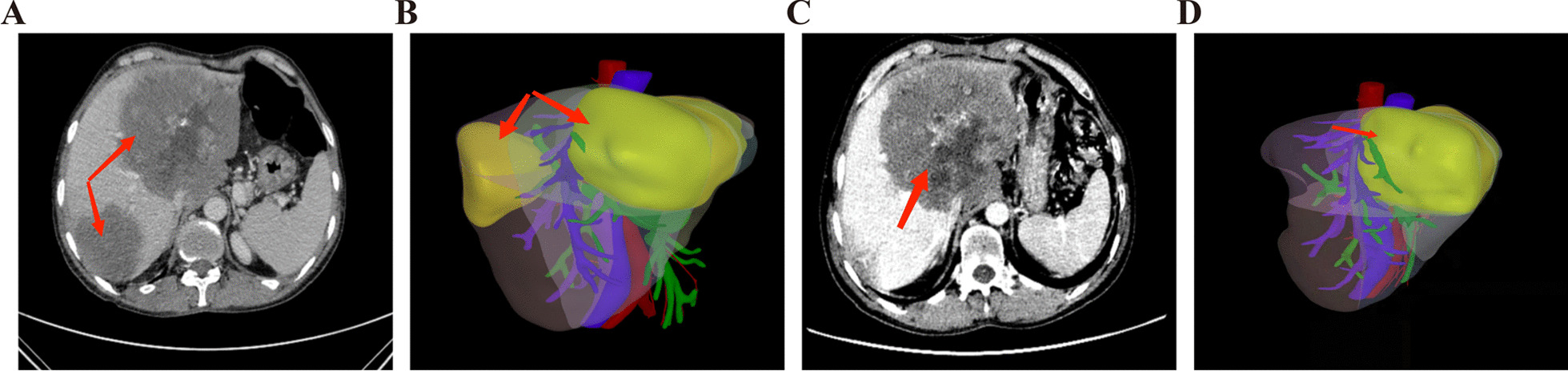
After TSH procedures, TSH3 patient, with FLRV/TLV > 30%, was able to receive ELRA procedures. **A** the first stage preoperative CT image; **B** the first stage preoperative digital 3D reconstruction of liver (FLRV/TLV < 30%); **C** CT image before the second stage operation; **D** The second stage preoperative digital 3D reconstruction of liver (FLRV/TLV > 30%)

The marginal hepatic AE lesions were removed at the first stage surgery as planned. The oblique incision under the right costal margin made it possible to enter the abdominal cavity layer by layer. Liver and abdominal cavity were carefully explored to determine the invasion scope of AE lesions. The lateral liver was carefully dissociated, and the sickle ligament was selectively disconnected as needed. In order to avoid tissue adhesion and reduce unnecessary dissociation during the second stage surgery, the first hilar was anatomically exposed. Then, a medium ventricular drainage tube was suspended at the hepatoduodenal ligament to expose the hepatic segment or hepatic lobe where AE lesions were located. The first hilar was blocked intermittently, and the hepatic parenchyma was disconnected by the combination of clamp and cavitron ultrasonic surgical aspirator (CUSA) right before invaded blood vessels and biliary ducts were gradually ligated. Ducts smaller than 0.3 cm were clamped by titanium clamp while ducts larger than 0.3 cm and smaller than 1.0 cm were ligated with suture. Additionally, ducts larger than 1.0 cm were sutured after disconnection. Finally, invaded portal vein and hepatic artery branches that enter the lesion to be removed were disconnected, whose reserved end was sutured with 5.0 polypropylene (Prolene) sutures. When the ratio of FLRV to TLV was greater than 30% (greater than 40% in patients with chronic hepatitis), second stage radical surgery was performed. Representative imaging pictures before the first stage operation and the second stage operation were displayed in Figs. 4, 5.

### Albendazole medication

Following recovery of liver function after the first stage operation, all patients started to take albendazole (10-15 mg/kg/d in 2 divided doses) until the second stage operation [15]. During the drug treatment, the blood routine and liver function tests of these patients were monitored. In case of any abnormality, the blood monitoring was rechecked after one to two weeks of drug withdrawal, and albendazole can be taken again after recovery.

### Follow‑up protocol

Follow-up was performed by consulting the outpatient and inpatient medical records after the first stage operation or through direct telephone contact. The follow-up mainly included the monthly liver function [total bilirubin (TBil), alanine aminotransferase (ALT), aspartate aminotransferase (AST), albumin (Alb), gamma-glutamyl transpeptidase (GGT)] and liver CTA examination results of PVE patients, the monthly liver function examination of TSH patients, and the monthly liver CTA examination results from the third month after the first stage surgery. According to the liver CTA imaging results, the FLRV was dynamically evaluated. If the preliminary assessment showed that the hyperplasia was good, digital 3D reconstruction was performed to further calculate the FLRV. Postoperative complications, as another important follow-up item, were recorded with Clavien-Dindo grading system [16].

### Statistical analysis

The data were analyzed by statistical product and service solutions (SPSS) software (ver. 19.0 for Windows; SPSS Inc, Chicago, IL, USA). In the statistical description of quantitative data, if the data met the normality, mean and standard deviation were selected; otherwise, median and interquartile range (IQR) were selected. For the comparison of paired designs, when the difference *d* met the normality, the paired *t* test was selected; otherwise, the paired rank sum test was selected. *P* < 0.05 was considered statistically significant.

## Results

### Basic clinical information of the participants

In this study, 19 hospitalized patients suffering from confirmed advanced hepatic AE accompanied with insufficient FLRV were enrolled. All the patients used to live in the epidemic areas. There were nine males and ten females, whose ages ranged from 21 to 68 years with the average age of (39.42 ± 12.47) years plus with the median age of 37 years. The mean body mass index (BMI) stood at (21.52 ± 1.80) Kg/m^2^. The mean indocyanine green retention rate after 15 min was (7.75 ± 3.80) %. Fifteen cases had huge irregular liver lesions, and the other four had multiple liver lesions of different sizes, including three with two lesions and one with three lesions. Among the 19 patients, four had chronic hepatitis (21.05%), two had lung metastasis (10.53%), one had brain metastasis (5.26%), and one had septal angle metastasis (5.26%). Nineteen patients had different degrees of abdominal pain, and four of them (21.05%) were accompanied by skin and sclera jaundice. According to the imaging analysis, all patients had varying degrees of biliary and vascular invasion. The liver functions fell into Child Pugh Grade A in 15 cases and Child Pugh Grade B in four patients for whom, PTCD was used to improve their liver functions before the operation due to the combination of obstructive jaundice.

### Operative treatment

Among the 19 patients, 15 patients received PVE treatment. According to the extent of lesion invasion and the size of the remaining normal liver, ten cases of right portal vein embolism, one case of right anterior portal vein embolism, and four cases of left portal vein embolism were performed. Among them, 12 patients underwent secondary radical surgery after PVE, including two cases of extended right hepatectomy, one case of right hepatectomy, two cases of extended left hepatectomy, and seven cases of ELRA. The median interval between two operations of the 12 patients stood at 3.95 months (IQR 2.56–4.44). The remaining three patients did not receive surgical treatment: one failed to follow the discharge doctor's advice due to personal reasons, and thus did not receive preoperative evaluation again as well as surgical treatment; another two failed to receive second stage operation due to economic reasons despite the proper FLRV.

The remaining four patients received TSH treatment, and the first stage operation was performed differently according to the scope of lesion invasion, as well as the number and size of lesions. The first patient underwent hepatic left medial sectionectomy combined with right posterior sectionectomy, the second hepatic left lateral sectionectomy, the third hepatic right posterior sectionectomy and the fourth left hepatectomy. FLRV of four patients increased significantly after the first stage operation. However, since the initial lesion seriously invaded the first and second hepatic hilum, radical resection could not be performed in vivo. Therefore, four patients were treated with ELRA for the second stage surgery, achieving favorable clinical effects. The median interval between two operations stood at 7.53 months (IQR 3.52–10.30).

### Comparison of changes of liver function and liver volume before and after PVE

In terms of the liver function indicators before PVE (preoperative indicators) and after PVE (pre discharge indicators), there was no significant difference in TBil, ALT, AST, Alb and GGT before and after PVE in all patients (*P* > 0.05).

In terms of the liver volume before (measured before surgery) and after PVE (measured in one to three months after PVE, if the FLRV failed to meet the surgical indication, the interval time between the two operations would be extended), the TLV and the lesions volume before and after PVE had no significant difference (*P* > 0.05). However, the difference was statistically significant (*P* < 0.05) in FLRV and FLRV/TLV. Details were shown in Table 1.

**Table 1:** Changes of liver function indexes and liver volume before and after PVE (M(IQR))

Variables	Before PVE	After PVE	*Z* value	*P* value
TBil (μmol/L)	12.99 (7.94–28.27)	16.06 (10.84–31.08)	− 0.973	0.331
Alb (g/L)	36.65 (35.59–38.55)	36.48 (33.17–39.78)	− 0.94	0.925
AST (U/L)	28.75 (23.82–59.60)	40.13 (24.28–102.05)	− 1.412	0.158
ALT (U/L)	43.92 (23.08–78.78)	59.13 (23.91–147.74)	− 1.852	0.064
GGT (U/L)	159.65 (69.06–273.95)	107.00 (56.03–153.35)	− 0.664	0.507
TLV (cm^3)^	1910.76 (1453.60–3218.46)	1889.50 (1646.28–2792.19)	− 0.785	0.433
LV (cm^3)^	750.05 (335.73–1403.56)	582.41 (344.85–1087.09)	− 0.847	0.397
FLRV (cm^3)^	471.34 (306.92–685.78)	794.90 (636.08–1188.28)	− 3.296	0.001^*^
FLRV/TLV (%)	23.49 (17.61–27.82)	38.81 (35.78–46.93)	− 3.296	0.001^*^

*PVE* portal vein embolization, *TBil* total bilirubin, *Alb* albumin, *AST* aspartate transaminase, *ALT* alanine aminotransferase, *GGT* gamma-glutamyl transpeptidase, *TLV* total liver volume, *LV* lesions volume, *FLRV* future liver remnant volume

### Comparison of changes of liver function and liver volume before and after the first stage operation of TSH

The liver functions of patients with TSH before and after the first stage operation (pre discharge index) showed no significant changes in TBil and Alb, but AST, ALT and GGT decreased significantly.

In four patients with TSH, the median FLRV (measured before operation) before the first stage operation was 492.05 cm^3^ (IQR 328.28–677.38), the median TLV was 1951.42 cm^3^ (IQR 1499.55–2051.45), and the median FLRV/TLV was 29.88% (IQR 18.96–33.13). The median FLRV after the first stage operation (the FLRV was generally measured three months after the operation, and the interval would be extended if the indication for the second radical operation was not reached after the measurement) was 779.75 cm^3^ (IQR 703.50–952.16), the median TLV was 1648.57 cm^3^ (IQR 1256.03–2069.90), and the median FLRV/TLV was 52.01% (IQR 42.33–57.00). The FLRV was significantly larger than before. Details were shown in Table 2.

**Table 2:** Changes of liver function indexes and liver volume in 4 patients treated with TSH before and after the first surgery (M(IQR))

Variables	Before the first surgery	After the first surgery
Tbil (μmol/L)	9.29 (6.07–24.34)	11.93 (6.55–21.24)
Alb (g/L)	36.70 (34.93–46.80)	36.78 (30.49–42.50)
AST (U/L)	39.95 (17.90–67.55)	18.82 (18.05–36.96)
ALT (U/L)	60.50 (12.00–141.00)	17.32 (15.54–38.52)
GGT (U/L)	123.00 (28.50–392.70)	63.19 (28.20–92.50)
TLV (cm^3^)	1951.42 (1499.55–2051.45)	1648.57 (1256.03–2069.90)
LV (cm^3^)	667.53 (423.63–1034.05)	518.15 (202.63–1008.49)
FLRV (cm^3^)	492.05 (328.28–677.38)	779.75 (703.50–952.16)
FLRV/TLV (%)	29.88 (18.96–33.13)	52.01 (42.33–57.00)

*TSH* two-stage hepatectomy, *TBil* total bilirubin, Alb albumin, *AST* aspartate transaminase, *ALT* alanine aminotransferase, *GGT* gamma-glutamyl transpeptidase, *TLV* total liver volume, *LV* lesions volume, *FLRV* future liver remnant volume

### Follow-up data and survival of the participants

Of the 15 patients who received PVE, one was lost to follow-up and was not readmitted for further surgery. Another two were prevented by financial burdens despite sufficient FLRV for the second stage surgery. The rest 12, with sufficient FLRV, underwent the second stage radical resection, including ELRA in seven patients and hepatectomy in five patients, with the median interval of 3.95 months (IQR 2.56–4.44). Among the seven patients who underwent ELRA, six developed Clavien-Dindo IIIa complications and one developed Clavien-Dindo I complication after surgery. Among the five patients underwent hepatectomy, one patient developed Clavien-Dindo IIIa complication and four patients developed Clavien-Dindo I complications. All complications were either ascites or pleural effusion. Fourteen patients followed up for a median interval of 4.10 months (IQR 2.61–4.64).

For the four patients who underwent TSH, the median interval time after the first stage hepatectomy stayed at 7.53 months (IQR 3.52–10.30). There were no severe postoperative complications, and hepatic lobe hyperplasia was obvious. After readmission, the ratios of FLRV to TLV were greater than 30% in all patients, who then further underwent ELRA for the second stage radical surgery. Four patients developed Clavien–Dindo IIIa complications, being either ascites or pleural effusion.

## Discussion

Hepatic AE, known as worm cancer, has high endemicity in western China, with Tibet, Qinghai, Xinjiang and Gansu included, and continues to be a serious public health issue, causing heavy economic burden to the local farmers and herdsmen [17, 18]. As reported, AE patients show no typical symptoms at earlier stages, but many suffer a mortality rate of 90% after 10 years if left untreated [18]. In 2012, with the World Health Organization (WHO) listing echinococcosis as one of the 17 neglected diseases to be controlled or eliminated by 2050, more attention has been given to hepatic AE [19]. Despite radical resection as the only effective treatment, the disease often has progressed to advanced hepatic AE when the patient is admitted, which is characterized by a single or multiple giant alveolar echinococcal lesions that seriously invade into the important vascular structure of the hepatic hilum, resulting in complex anatomical spatial relationships [20]. Consequently, most patients lose the opportunity to receive radical surgical resection. Moreover, if the problem of insufficient FLRV after surgery cannot be fundamentally solved, liver failure and even death may occur after radical resection [21–23]. Liver transplantation can serve as the ultimate solution for advanced hepatic AE patients with insufficient FLRV. However, the shortage of liver source and long-term use of anti rejection drugs make it unable to be widely developed. Therefore, a safe and effective liver lobe proliferation promotion technique is urgently needed to ensure the proliferation of the remaining functional liver and radical surgery. In regard to this issue, our center adopts PVE and staged hepatectomy for advanced hepatic AE patients.

The key point of remnant liver volume evaluation lies in the accurate measurement of remnant liver volume before surgery. [24]. Individualized hepatic reconstruction systems and 3D imaging are vital for accurately removing AE lesions, which not only reduces unnecessary damage to hepatic AE patients, but also helps surgeons to quantify the liver [25]. Meanwhile, quality of the remaining liver is equally important in clinical settings. It has been reported that when the hepatic lobe is proliferating, accompanied complications, such as obstructive jaundice and biliary acid retention, may cause hepatic injuries [26]. Clinically, most advanced hepatic AE patients are usually accompanied by biliary obstruction. Therefore, preoperative biliary decompression is particularly essential. In this present study, four patients presented as abnormal liver functions before PVE due to obstructive jaundice, which were dealt with PTCD. After their liver functions returned to the normal level, PVE was performed to ensure the quality and quantity of proliferative liver, which also laid better foundation for the second stage surgery. Recent studies have demonstrated that preoperative PVE was efficient for reducing the risks of postoperative liver failure caused by insufficient FLRV, and it played significant roles in promoting proliferation of the unaffected liver [27–29]. Strikingly, our results showed that there was no significant difference in the changes of liver functions before and after PVE, suggesting that PVE application in patients with advanced hepatic AE did not increase the hepatic burden. Instead, this technique opened up a novel modality to better solve FLRV insufficiency for such patients.

Literatures in recent years have shown that PVE and TSH have made some progress in the application of hepatic malignancies [30–32], but there are few studies on advanced hepatic AE. It should be emphasized that hepatic AE and hepatic malignancies have different biological characteristics. For hepatic malignancies with insufficient FLRV, the biggest problem of liver lobe proliferation techniques lies in that the tumor progresses rapidly in the waiting period, which makes about 25% of patients unable to undergo reoperation [33]. Therefore, the waiting time was short, generally four to six weeks. However, hepatic AE is a benign disease with slow growth and a chronic course of nearly ten years. After the first stage operation, the patients had sufficient waiting time windows. Literature reports show taking albendazole before and after surgery may benefit patients with echinococcosis [34]. In fact, albendazole tablets can delay the lesion progression, and thus win time for further operation [35, 36]. Therefore, patients receiving PVE or the first stage surgery of TSH treatment in this study began to take albendazole tablets after the liver function recovered to normal until the FLRV was tolerable for the second stage operation. In order to prevent postoperative recurrence, all patients continued to take albendazole according to the above method for two years after the second stage operation [37–39].

In this current study, the median interval time between PVE and planned second staged radical surgery in 14 patients stood at 4.10 months, and all of them received effective treatment. Postoperative FLRV increased from 471.34 cm^3^ to 794.9 cm^3^; whereas there were no significant alterations in the lesions volume (750.05 cm^3^ VS 582.41 cm^3^). Importantly, the median ratio of FLRV to TLV increased from 23.49 to 38.81% with a median increase rate of 4.49% per month. Compared with hepatic malignancies, PVE allowed patients with advanced hepatic AE who had lost opportunity for radical surgery to re-obtain chances to undergo radical surgery, and served as a better choice. Of note, our study preliminarily suggested that PVE was optimal for hepatic AE patients without portal hypertension and portal vein thrombosis as well as those whose AE lesions were located in the middle hepatic lobe or one side of the liver [40]. The clinical practice of TSH in the treatment of advanced multiple hepatic AE lesions originated from the treatment strategy of TSH in colorectal cancer patients with hepatic metastasis [41]. In our study, there were multiple AE lesions in four patients, with the first, second hilar as well as inferior vena cava hepatic segments seriously invaded. Thus, it was difficult for radical hepatectomy in vivo, and performing ELRA was also a tough task for surgeons due to insufficient FLRV. Through 3D imaging, three-dimensional structures of liver, AE lesions and the vascular system were constructed, and FLRV was calculated based on its calculation functions. Before the first stage surgery, the median FLVR, TLR and the ratio of FLRV to TLR were respectively 492.05 cm^3^, 1951.42 cm^3^ and 29.88%. The median interval time after the first stage resection for these patients was 7.53 months. Fortunately, the median FLRV, TLV and the ratio of FLRV to TLR significantly increased and were respectively 779.75 cm^3^, 1648.57 cm^3^ and 52.01% after the first stage surgery. Then, ELRA was successfully performed for all patients, who were discharged after obvious improvement. Altogether, it was preliminarily suggested from our study that TSH should not be restricted to the traditional surgery, and combining it with ELRA was also safe and feasible to treat advanced hepatic AE with multiple lesions and severe invasion of the first as well as second hilar. As a promising technique for promoting hepatic lobe hyperplasia, TSH was more suitable for patients with multiple hepatic AE lesions, which was slightly different from PVE. It should be noted that the invasive range, size, number, remaining normal liver volume, the liver segment to be embolized by PVE, and the range of lesions to be removed for the first stage by TSH are different for each patient. Therefore, the interval time after PVE or TSH is also different for each patient. Patients were empirically instructed to review the liver CTA and 3D imaging to assess the growth of the remaining normal liver volume one to three months after discharge from the hospital after the first stage operation, which would help determine whether to tolerate radical resection or ELRA. In case the remaining normal liver volume after measurement was not enough for the second surgical treatment, the interval would be extended. Patients failing to follow the doctor's instruction after discharge or failing to be admitted to the hospital in time for review due to economic reasons also made the interval relatively longer. Fortunately, the patients' conditions were stable during the waiting period, and the second stage surgical treatments were not affected. In this study, the longest interval after the first stage hepatectomy for the two patients who needed ELRA treatment reached up to ten months. With significantly increased FLRV and no significant progress in AE lesions, ELRA was successfully implemented to guarantee survivals of the patients. Additionally, ALPPS, as another hepatic lobe hyperplasia technique, can promote rapid proliferation of the healthy side liver. However, compared with TSH, ALPPS has higher incidences of postoperative complications and mortality. There have been cases of successful application of ALPPS in the treatment of advanced hepatic AE, but its surgical indication needs to be further studied and clarified [42].

The FLRV of 14 patients undergoing PVE proliferated well enough for the second stage radical surgery. The average FLRV increase rate stood at 4.49% per month, compared with no obvious alterations in the lesion volume (750.05 cm^3^ VS 582.41 cm^3^) and total liver volume (1910.76 cm^3^ VS 1889.50 cm^3^), indicating that proliferation of remnant liver volume was materialized without further stimulating growth of AE lesions and thus achieving expected clinical outcomes. Moreover, in the four patients receiving TSH, small AE lesions were removed at the first stage surgery to ensure better proliferation of the healthy side liver, which greatly retained the normal functional liver. Therefore, the preoperative median FLRV at the second stage was significantly higher than that at the first stage (779.75 cm^3^ VS 492.05 cm^3^), which brought new possibilities for patients whose AE lesions may not be radically removed through one stage surgery. In line with the developmental concept of precision hepatic surgery, TSH not only ensured the surgical safety, but also opened up novel ideas for surgical modalities of multiple AE lesions, especially of AE lesions located in both hepatic lobes.

Previous literature has reported the use of PVE or TSH on patients suffering from hepatic AE with insufficient FLRV in a small sample size or in a limited form of case report. Shen et al. have reported that seven patients with advanced multiple giant hepatic AE were treated through TSH, which effectively reduced the operation risk and achieved radical resection [43]. Similarly, these seven patients had at least two lesions and the FLRV was less than 30% of the total liver volume. In the first stage operation, all patients had partial lesions removed through hepatectomy. When FLRV reached the surgical standard three months later, the second stage operation was performed after which they all took albendazole tablets for a long period of time. However, method used for the second stage operation was different from ours. Excluding patients with portal vein trunk or inferior vena cava invasion, only hepatectomy was performed during the second stage operation to remove the remaining lesions. The four patients in our study, however, performed ELRA to remove the remaining lesions in the second stage operation, yielding good clinical results. Our study preliminarily showed that TSH was not only safe and feasible, but also enjoyed broader surgical indications. However, the small number of cases need to be further confirmed by larger sample studies. In addition, Sun et al. also reported a case of TSH combined with PVE to effectively promote the growth of FLRV in patients with advanced hepatic AE [44]. According to preoperative evaluation, TSH was first performed to remove AE lesions in the left lobe of the liver. The patient then received PVE resulting in a significant increase in FLRV. The AE lesions of VII and VIII segments were subsequently removed in the second stage operation. It must be noted that the surgical strategy of PVE combined with TSH is innovative, which is also the most significant difference from our study.

Preliminary experience has been gained in the application of hepatic lobe hyperplasia techniques in advanced hepatic AE reported in this study, but there are still some limitations. First of all, this study falls into a retrospective analysis, which requires queue study to further explore the clinical effects of the application of the liver lobe hyperplasia promotion techniques in the advanced hepatic AE. Secondly, due to the low incidence rate of hepatic AE, the slow growth characteristics of the lesions and the long duration of the disease, the number of patients with advanced AE is relatively small. Thus, application of hepatic lobe hyperplasia techniques in the treatment of advanced AE is still at the clinical exploration stage in recent years. However, the 19 cases in this study are currently the largest sample size of a study of hepatic lobe hyperplasia techniques applied to advanced hepatic AE. Moreover, 19 patients were all from our center, so we need to make further experience summary on the clinical efficacy of hepatic lobe hyperplasia techniques in a prospective, multi center study in the future.

## Conclusion

PVE and TSH can effectively promote the growth of FLRV in patients with advanced hepatic AE with insufficient remnant liver volume for further radical resection.

## Data Availability

The datasets generated and analysed during the current study are not publicly available due to restrictions on ethical approvals involving patient data and anonymity but are available from the corresponding author on reasonable request.
